# Observations on Dr. Harrison's Bill

**Published:** 1811-03

**Authors:** 


### *To the Editors of the Medical and Physical Journal.*

GENTLEMEN,

A Correspondent, in the last number of your Journal, having undertaken the defence of Dr. Harrison's projected Bill, I am induced to trouble you with a few cursory observations on a scheme, which has always appeared to me very inadequate to the end proposed, and respecting the good effects of which, if it ever passed into a law, I must still confess myself more than 'sceptical,' notwithstanding the 'explanation' which its present advocate has offered.

In the first place, however, I must beg leave to advert to the very singular attempts which he has made, to repress all free inquiry into the merits of the question, by his mysterious hints concerning the '*subsequent matters in contemplation*,' and by his still more strange insinuation, that as the bill has been 'framed by *a Barrister of the first eminence in this department of law*,' no person should 'venture to oppose or condemn' any of its provisions, *without first taking the opinion of counsel*, or, to use the writer's own words, without 'the sanction of a *legal* practitioner.' Now, without any disparagement to this 'Barrister of the first eminence,' I should conceive that medical men were in general as well qualified to judge of the abuses in their profession, and to suggest the appropriate remedies, as any of the gentlemen learned in the law could possibly be: and if a legislative scheme be radically bad or defective, as I believe is the case with the one now under consideration, it would be a small merit in my estimation, that it was the production of the united industry of all the *Barristers* in the kingdom, or that, after it was carried, some 'subsequent matters' were 'in contemplation.' Nor do I think it necessary to incur the expense of 'legal' advice, in order to have the privilege of stating 'difficulties' which must, I think, be obvious to every man of common reflection.

Complying with the suggestion of H. R. I shall endeavour to 'confine *my* remarks to what is contained in the bill itself,' and am, in truth, contented to try it on its own merits; for there is quite enough in it to prove its futility and absurdity. What, then, does this great bill provide? Why, that henceforth all physicians, surgeons, apothecaries, midwives, chemists, horse-doctors, bone-setters, &c. shall enter their names in one grand register, for which they shall pay a certain sum, and place their names upon the doors doors of their respective dwellings; that the powers of all existing medical corporations shall be confirmed, and in some respects increased to a most dangerous extent; that moreover the magistrates at the Quarter Sessions shall be authorized to licence every ignorant pretender to medical skill who thinks fit to apply: and that the sale of stamped medicines shall be countenanced and legalized "*in omnia secula seculorum.*"—Such are the leading features of the "Bill for the improvement of the Medical and Surgical, and Veterinary Sciences, and for regulating the practice thereof."—Such the glorious off-spring of the lucubrations of the 'Barrister of the first eminence *in this department of the law.*' But are there no clauses by which the mischiefs that must arise from thus confounding the regular physician and surgeon with the '*Ambubiarum collegia, pharmacopolæ, et hoc genus omne*,' would be counterbalanced; which would secure the public from the depredations that might be committed by impostors acting under the sanction of country justices; and which would check, if they did not altogether repress, the inroads of quackery? On the contrary, every thing seems to have been studiously contrived with the view of degrading all that is great, and learned, and respectable, in the profession, of consolidating every abuse that exists, and of perpetuating every irregularity of which Dr. Harrison and his fellow-reformers so loudly complain. These gentlemen, it is true, profess to be actuated by very different motives, and I believe them to be well meaning men; but if we grant them the merit of sincerity, it must be at the expense of their judgment and sagacity. That I have not misrepresented the tendency of their bill will be evident, I think, to any one who takes the trouble to examine it with attention: it is only difficult to imagine what benefits those who framed it expect to accrue from it; for notwithstanding all the declamations of Dr. Harrison about the 'reform of abuses,' the suppression of 'the most dangerous empirics,' and the 'immediate advantages to society at large,' I am unable to perceive, that he or his friends have one clear idea on the subject.

But to return to your correspondent, in whom this measure has found so warm a defender: 'Provision,' he observes, 'is made in the bill, not only for the passing generation *to continue their functions unmolested*, BUT TO CONFIRM THEM IN THE LEGAL ENJOYMENT OF THEIR SEVERAL PRACTICES; consequently the plan will be *highly beneficial* to the present establishment. To the quack and empirical pretender undoubtedly it will be 'highly beneficial,' as it tends 'to confirm them in the *legal* enjoyment of their several practices:' not so to the physician and surgeon of education, who want no such confirmation, and who certainly will not be raised in the public opinion by being classed and 'registered' with the scum of the profession. 'The bill,' however, we are told, 'seems in one material instance to have been misunderstood, and in consequence to have been unjustly abused. An opinion seems to prevail,' your correspondent continues, 'that the bill will forcibly restrain physicians, surgeons, and apothecaries to their respective lines. No such thing was ever in contemplation. Dr. Harrison, *to tranquillize the apprehensions of the profession*, declares in his pamphlet, "in order to remove the fears, and correct the mistakes of some people, it may be proper to observe, that the bill does not attempt to limit the sphere of medical duty by coercive statutes. *Practitioners will be left under it at full liberty to use their talents according to their own discretion.*" Most satisfactory assurance! all the inconveniencies, jealousies, and enmities, that spring from the jostling of the different classes of practitioners, are accordingly to be cherished by this wonder-working bill: the surgeon may encroach *ad libitum* on the province of the physician; the compounder of medicines may have the whole range of the art; and the illustrious fraternity of quacks may 'use their talents according to their own *discretion!*' 'It will however oblige all *future* medical men to pass through a suitable course of study, and undergo examinations for the particular branch, or branches of the profession, into which they are admitted.' This is so far good: but surely the present generation is of some consequence, and ought to have come in for a share of the happiness which the ages yet unborn are to enjoy, instead of being left, as before, to suffer from 'irregularities of the most disgraceful and dangerous kind.'

'*Qui trop embrasse, peu estraint*,' said Grangousier to his prisoner Toncquedillon. To the labours of the Association the remark is strikingly applicable. Wishing to reform every thing, they mend nothing; wishing to please every body, they satisfy no one with their scheme: they leave matters just where they found them, or, rather, they leave them, supposing their plan to receive legislative sanction, in much worse condition; for a bill of the nature of that now proposed would give rise to endless embarrassment and confusion in the practice of the art. Your correspondent will, perhaps, insinuate, that I condemn it too hastily, and without sufficiently examining its different provisions. I can assure him, however, that I have studied it with considerable care; that I have read all the principal publications on the subject; and that if I do not speak of the measure in such respectful terms as its propounders might desire, it is only because because I find it replete with mischief, while its good effects are to my mind extremely equivocal. If your correspondent will point out in what manner any one beneficial consequence must necessarily flow from his favourite bill, that will not be counterbalanced by an attendant evil, I shall be ready to moderate my censure; but he must give me something more than vague declamation on the abuses in practice, and general assertionas to the efficacy of an act of parliament in correcting and removing them.

I am, Gentlemen,
Yours, &c.

A. H.

*London, Feb.* 11, 1811.

